# deepTAD: an approach for identifying topologically associated domains based on convolutional neural network and transformer model

**DOI:** 10.1093/bib/bbaf127

**Published:** 2025-03-25

**Authors:** Xiaoyan Wang, Junwei Luo, Lili Wu, Huimin Luo, Fei Guo

**Affiliations:** School of Software, Henan Polytechnic University, 2001 Century Road, Jiaozuo 454003, China; School of Software, Henan Polytechnic University, 2001 Century Road, Jiaozuo 454003, China; School of Software, Henan Polytechnic University, 2001 Century Road, Jiaozuo 454003, China; School of Computer and Information Engineering, Henan University, North Section of Jinming Avenue, Kaifeng 475001, China; School of Computer Science and Engineering, Central South University, 932 Lushan South Road, Changsha 410083, China

**Keywords:** topologically associating domains, convolutional neural network, transformer, three-dimensional genome

## Abstract

Motivation: Topologically associated domains (TADs) play a key role in the 3D organization and function of genomes, and accurate detection of TADs is essential for revealing the relationship between genomic structure and function. Most current methods are developed to extract features in Hi-C interaction matrix to identify TADs. However, due to complexities in Hi-C contact matrices, it is difficult to directly extract features associated with TADs, which prevents current methods from identifying accurate TADs. Results: In this paper, a novel method is proposed, deepTAD, which is developed based on a convolutional neural network (CNN) and transformer model. First, based on Hi-C contact matrix, deepTAD utilizes CNN to directly extract features associated with TAD boundaries. Next, deepTAD takes advantage of the transformer model to analyze the variation features around TAD boundaries and determines the TAD boundaries. Second, deepTAD uses the Wilcoxon rank-sum test to further identify false-positive boundaries. Finally, deepTAD computes cosine similarity among identified TAD boundaries and assembles TAD boundaries to obtain hierarchical TADs. The experimental results show that TAD boundaries identified by deepTAD have a significant enrichment of biological features, including structural proteins, histone modifications, and transcription start site loci. Additionally, when evaluating the completeness and accuracy of identified TADs, deepTAD has a good performance compared with other methods. The source code of deepTAD is available at https://github.com/xiaoyan-wang99/deepTAD.

## Introduction

High-throughput chromosome conformation capture technology (Hi-C) is usually used to study the 3D spatial structural organization of chromatin in nuclei, enabling the capture of interactions between chromosome segments within genome-wide units [1]. Hi-C paired reads have revealed a range of structural features, including A/B compartments [2], topologically associated domains (TADs) [3], and chromatin loops [4, 5]. Among them, TADs are regions of the genome with continuous self-interactions that have a significantly higher frequency of internal contacts than surrounding regions. These domains typically range in size from 200 kilobases (kb) to 5 megabases (Mb) [6], contain abundant genomic elements [7, 8], and exhibit a high degree of conservation across different species and cell types. In some studies, TADs have been demonstrated to play a pivotal role in gene expression regulation [9–11], cell differentiation and development [12], and the occurrence of diseases and tumors [13–16]. From a biological perspective, accurate prediction of TAD boundaries is essential for understanding enhancer–promoter interactions, which are mediated by chromatin loops an chored at these boundaries. These interactions ensure that enhancers act specifically on their target genes within the same TAD, preventing the misregulation of genes outside the boundaries. For instance, [17] demonstrated that disruption of TAD boundaries can result in ectopic enhancer-promoter interactions, leading to the misexpression of autism spectrum disorder-related genes. Similarly, boundary-crossing enhancers can aberrantly activate oncogenes, contributing to tumorigenesis, while mutations in enhancers or other non-coding regions within TADs may disrupt normal gene regulation [18]. Therefore, developing a method that can quickly and accurately identify TADs is crucial for revealing the mysteries of the 3D structure of chromosomes and studying their impact on diseases.

Several computational methods have been developed to analyze Hi-C data and identify TADs with high accuracy and reliability. Most TAD detection methods directly use the features and patterns in the Hi-C contact matrix to design objective functions to solve this problem [19–23], whereas others rely on graph partitioning [24–26], clustering algorithms [3, 27–31] and machine learning techniques [32]. Dixon *et al*. used the directionality index (DI) [3] to detect bins with significant upstream and downstream interaction differences as TAD boundaries, applying a hidden Markov model to define TADs. Inspired by this, various computational methods have emerged to detect TAD boundaries and infer TAD structures. The deDoc method [24] is a graph partitioning method, i.e. used to generate an encoding tree, i.e. greedily combined by minimizing the global uncertainty of the Hi-C graph until continuous leaf nodes are partitioned into TADs. TopDom [23] analyzes weak interactions at TAD boundaries using an adjustable window size to limit interaction frequency calculations. It extracts statistical data from the Hi-C interaction matrix, identifying local minima in interaction strength as potential TAD boundary points. EMTAD [33] utilizes the empirical mode decomposition method to enhance Hi-C interaction matrix data. This method adaptively decomposes Hi-C data into the sum of multiple feature mode functions to identify TADs from the optimized data. CATAD [34] is a TAD identification method that relies on a core-attachment structural model that uses local density and cosine similarity to detect the core of a TAD and boundary insulation to determine attachments. LPAD [26] extracts node correlations from the global interactions of chromosomes and then constructs an undirected graph *via* the Hi-C contact matrix. Communities are discovered, and TADs are generated through label propagation.

In the above methods, TADs are primarily identified by describing the bins that are at the TAD boundaries or clustering them based on their empirical distributions within the TAD. Existing conventional methods for TAD identification typically focus on either local chromatin features (e.g. TopDom [23], CaTCH [35]) or long-range interactions (e.g. DI [3]), but rarely integrate both. This separation often leads to limitations in detecting nested TAD structures and reduces the ability to identify broader patterns of chromatin organization. In addition, computational efficiency is challenging for some methods, such as Arrowhead and TopDom [23], particularly when applied to large genomes or high-resolution datasets. These limitations limit their practicality for genome-wide TAD identification, especially in species with complex genomes or studies requiring high-throughput analyzes. In contrast, existing deep learning methods predominantly rely on convolutional neural networks (CNNs), such as TADL [36] and TAD_Boundary_Detector [37], or traditional machine learning models, such as StackTADB [38]. While CNNs are well-suited for extracting local spatial features from chromatin interaction data, they often fail to capture long-range dependencies, essential for understanding hierarchical chromatin structures.

To overcome these limitations and improve the accuracy of TAD detection, we present a novel TAD identification method named deepTAD. In this method, deep learning and advanced feature extraction methods are used to transform the identification of TAD boundaries into a binary classification problem. The deep learning model in deepTAD uniquely integrates CNNs and transformers' strengths, representing a major advance over existing TAD prediction methods. CNNs effectively extract local spatial features from genomic data. However, they cannot model long-range dependencies, which are critical for understanding the hierarchical nature of chromatin interaction data. In contrast, with its self-learning mechanism, the transformer is excellent at capturing long-range dependencies but less effective at identifying fine-scale patterns [39]. Combining these two approaches, deepTAD creates a unified framework that captures local and global chromatin interaction features. And, deepTAD adopts the Wilcoxon rank-sum test to optimize boundary bins and cosine similarity to obtain hierarchical TADs. We evaluated the robustness of deepTAD across five cell types at different resolutions and evaluated its enrichment at TAD boundaries to validate its effectiveness. We also compare the performance of deepTAD with five other TAD callers, and our results demonstrate that deepTAD has some advantages in detecting TADs.

## Materials and methods

### Overview of deepTAD

**Figure 1:**
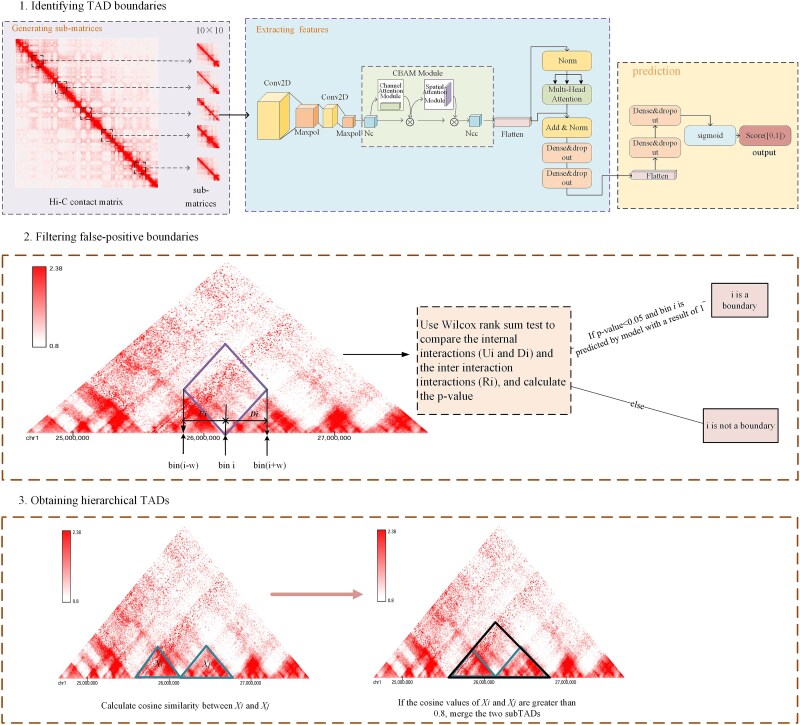
The workflow of deepTAD involves identifying TAD boundaries, filtering false-positive boundaries, and obtaining hierarchical TADs.

TADs in the Hi-C contact matrix are identified by deepTAD using the following three steps: (i) Identifying TAD boundaries: deepTAD first generates a sub-matrix for each bin. Next, for one sub-matrix, deepTAD extracts features using the CNN and the transformer model. Then, it judges whether the sub-matrix is a boundary. (ii) Filtering false-positive boundaries: for each candidate boundary, deepTAD adopts the Wilcoxon rank-sum test to filter false ones. (iii) Obtaining hierarchical TADs: deepTAD utilizes cosine similarity to assemble TAD boundaries and obtain hierarchical TADs. The overall workflow of deepTAD is shown in Fig. 1.

### Identifying TAD boundaries

#### Generating sub-matrices

Bins are contiguous segments or intervals of the genome, which have the same length. And the length of bin refers to resolution. In this study, the resolution defaulted to 25 kb.

deepTAD utilizes the Hi-C contact matrix *M* as input. The element *m_ij_* in *M* is the number of interactions between *i*th bin (Bin*_i_*) and *j*th bin (Bin*_j_*). For *Bin_i_*, a submatrix *SubM_i_* is extracted from *M,* where Sub*M_i_* = *M*[*i* − *4: i +* 5*, i* − 4*: i +* 5], encompassing the elements from rows (*i* − 4) to (*i* + 5) and columns (*j* − 4) to (*j* + 5). Hence, each sub-matrix is 10 × 10 in size. After this step, each bin corresponds to a sub-matrix.

#### Extracting features

In this step, TAD boundary features contained in a sub-matrix are extracted and analyzed. For one sub-matrix, deepTAD first utilizes a CNN model to capture local features. This CNN model contains two convolutional layers followed by a max pooling layer, which reduces the spatial dimensions of the features. The CNN's architecture allows deepTAD to effectively perceive local boundaries and maintain position invariance, enhancing its performance in feature extraction.

Then, to improve the accuracy of recognition, deepTAD employs a Convolutional Block Attention Module (CBAM) [40]. The CBAM applies the channel and spatial attention modules sequentially. Channel attention helps to enhance the feature representation of different channels, whereas spatial attention helps to extract key information at different locations in space. This allows more precise attention to key regions and features in the sub-matrix and improves identification accuracy. Using the CBAM attention mechanism, it is possible to reconstruct the feature map in the network. The goal is to increase the feature share of small targets and improve detection performance.

Previous steps are more focused on local feature extraction, aiming to capture small-scale changes in TAD boundaries. However, TAD boundary characteristics also can be reflected in global interaction variation. So, deepTAD has introduced the transformer model to further extract features. The transformer model constructed on the basis of the multihead self-attention mechanism can capture long-range representations of features. This mechanism enables the model to focus on the interrelationships between different parts when processing input data, thereby gaining a more comprehensive understanding of the global structure. After previous processing, deepTAD obtains a feature vector for the sub-matrix.

#### Prediction

Finally, the feature vector obtained is input into two fully connected layers for classification and prediction. Each fully connected layer is followed by a dropout layer, and the last fully connected layer uses a sigmoid function as the activation function. If the final output is >0.5, then the sample is considered a TAD boundary; otherwise, it is not a TAD boundary.

### Filtering false-positive boundaries

The Hi-C contact matrix often exhibits sparsity and includes noise, particularly in low-resolution datasets, which can obscure the signals necessary for accurate boundary identification. For the Hi-C contact matrix from high-resolution data, it also includes some noise. In addition, TAD boundaries are not always sharply defined. In many cases, the transition between two TADs is gradual rather than abrupt, making it difficult to determine the exact location of the boundary. This ambiguity creates uncertainty when distinguishing between positive and negative boundary bins. In addition, bins adjacent to a boundary may have partial boundary-like characteristics, further complicating their classification. Hence, deepTAD further adopts the method used in TopDom [23] to filter false-positive boundaries.

As described in TopDom [23], at the TAD boundary Bin*_i_*, the interaction between upstream and downstream bins (i.e. between two different TADs) is much smaller than the interaction among upstream bins or downstream bins. *U_i_* can represent the interaction among upstream bins of Bin*_i_*; *D_i_* can represent the interaction among downstream bins of Bin*_i_*. *R_i_* can represent the interaction between upstream bins and downstream bins. So, the Wilcoxon rank-sum test is utilized to judge whether there are significant differences between *U_i_* + *D_i_* and *R_i_*; those TAD boundaries with *P*-values exceeding .05 are filtered. When performing this filtering process, only a few boundaries predicted as positive samples are eliminated because they did not meet the significance requirements.

### Obtaining hierarchical TADs

On the basis of the previous steps, the TAD boundaries are obtained. Because each TAD consists of two boundary bins, the region between one boundary bin and the next boundary bin is defined as a TAD, in other words, if we have boundary bin set B: $\left[{r}_1,{r}_2,{r}_3,\dots, {r}_n\right]$, then the TAD set X is $\left[{r}_1,{r}_2\right]$, $\left[{r}_2,{r}_3\right]$,…,$\left[{r}_{n-1},{r}_n\right]$, i.e. X is [*X_1_*, *X_2_*, …, *X_n_*]. We adopt the method used in CATAD [34] to obtain hierarchical TADs. CATAD [34] employs cosine similarity to merge pre-cores, a process analogous to our task of detecting nested TAD structures, a small TAD corresponds to a pre-core. Similarly, SBTD [41] utilizes cosine similarity to assess interaction patterns between compartments, while TAD-Lactuca [42] uses cosine similarity to calculate vector-based histone modification signals at TAD boundaries and non-boundaries. These prior studies demonstrate that cosine similarity is effective in capturing relationships between genomic regions in TAD analyzes.

As described in CATAD [34], for sub-TAD ${X}_i$, if the cosine similarity between this sub-TAD and its upstream (downstream) adjacent sub-TAD ${X}_{i-1}$ (${X}_{i+1}$) is not greater than the threshold d*t*, then ${X}_i$ is considered to be a single TAD. For adjacent sub-TADs whose cosine similarity is greater than the threshold d*t*, the adjacent sub-TADs are merged so that they become a large TAD, which consists of a nested structure of TADs. If the cosine similarity value of two consecutive fields is less than the threshold, then they are not merged. Similarly, sub-TADs are judged for merging or not. The gap region is defined as a region of a certain *h* size in which the contact frequency is 0, labeled as a gap.

### Model training

#### Data acquisition and processing

Experimental datasets were downloaded from the Gene Expression Omnibus database (Login ID: GSE63525). Hi-C data from human GM12878, IMR90, K562, NHEK, HUVEC, and HMEC cell lines were used, and information on these datasets is detailed in Supplementary Table S1.

The Hi-C contact matrices for different samples are generated using juicer tools [43] with resolutions of 10, 25, 50, and 100 kb. To correct for biases in the data, the matrices were normalized using two widely used methods. The Knight-Ruiz (KR) normalization [44] minimizes systematic biases such as coverage variation and sequencing depth differences by iteratively equalizing the row and column sums of the contact matrix. The Vanilla-Coverage (VC) normalization [5] adjusts contact values based on the total number of interactions for each genomic locus, ensuring a more uniform distribution of interactions across loci. These normalization steps reduce biases inherent in Hi-C data, making the contact matrices more suitable for downstream analyzes such as TAD boundary detection.

In addition to the Hi-C data described above, ChIP-seq data for the human GM12878, IMR90, K562, NHEK, and HUVEC cell lines in the hg19 genome were downloaded from the UCSC Genome Browser (http://genome.ucsc.edu/). BED files for TSS and SINE in the hg19 genome were retrieved from the UCSC Genome Browser. Detailed information on how to access the above data is provided in Supplementary Table S2.

#### Constructing positive and negative samples

In TAD boundary identification, due to the lack of explicit ground truth labeling, the TAD boundaries identified by a single method may be affected by the bias of the methods themselves, while the number of boundaries jointly identified by the three methods is small, which is difficult to meet the sample size requirement for model training. Therefore, we finally chose the boundary shared by two methods as the positive sample. This strategy ensures the reliability of the labeling while ensuring the adequacy and diversity of the training dataset, thus striking a reasonable balance between data quality and quantity, which is crucial for effective model training.

So, for the Hi-C contact matrix, we select six well-performing TAD identification methods (CaTCH, CHAC, deDoc, DI, TopDom, and Arrowhead) to identify TAD boundaries. If one bin is considered a TAD boundary by two different methods, then this bin is identified as a positive TAD boundary, and other bins are identified as negative TAD boundaries. For a bin, we select its four upstream bins and five downstream bins and construct a 10 × 10 contact matrix from the Hi-C contact matrix. Hence, each bin corresponds to a 10 × 10 contact matrix.

When a bin is identified as a positive TAD boundary, its 10 × 10 contact matrix should contain various contact characteristics. In this 10 × 10 contact matrix, although the left bins interact frequently and the right bins also interact frequently, the interaction between the left bins and right bins is quite limited.

Consequently, if the bin is identified as a positive TAD boundary, then its 10 × 10 contact matrix is a positive sample. If the bin is identified as a negative TAD boundary, then its 10 × 10 contact matrix is a negative sample. Owing to the relatively small number of positive samples and the large number of negative samples, data augmentation was performed on the positive samples by rotating their corresponding 10 × 10 contact matrices 90° clockwise. This augmentation increased the number of positive samples, ensuring a more balanced dataset during model training. Additionally, negative samples four times the number of positive samples were selected to construct the negative samples.

#### Constructing training and validation samples

After contact matrices are generated, they are labeled. In this work, both the training and validation datasets were derived from the KR-normalized Hi-C contact matrix of the GM12878 cell line (HIC002) at a resolution of 25 kb, where chr1–12 is the training dataset, chr13–19 is the validation dataset, and chr20–22 is used as the testing dataset. Regarding chr X, it was not included in the testing phase of our study. This decision was made because chromosome X has unique structural and epigenetic characteristics. Including it in the testing phase could have introduced bias or confounding effects, potentially affecting the assessment of model performance. We implemented all the callers on a computer with a 24-core central processing unit (CPU) (Intel(R) Xeon(R) Platinum 8260 CPU @ 2.30 GHz). We used a single RTX 3090 video card for model training.

#### Hyperparameter settings

To achieve optimal model performance, we conducted extensive experiments to fine-tune the hyperparameters. Based on the results, we selected a random seed of 123, a learning rate of 0.0003, convolutional kernel sizes of (128, 3 × 3) for the first convolutional layer and (64, 3 × 3) for the second convolutional layer, and four attention heads for the multi-head attention mechanism. These parameters provided a balanced trade-off between precision, recall, and f1-score across different datasets. A detailed comparison of the hyperparameter configurations, including learning rates, kernel sizes, and number of attention heads, can be found in Supplementary Table S3, and experimental data for random seeds in Supplementary Table S4. This supplementary data demonstrate the robustness of the selected parameters in ensuring model stability and accuracy.

### Evaluation metrics

#### Average peak

The average peak [45] is a density measurement used to describe the frequency of occurrence of regulating elements near the TAD boundary. The calculation formula is (1):


(1) \begin{equation*} \text{Average}\ \text{peak}=\frac{1}{n}\sum_{i=1}^n{D}_i. \end{equation*}

Let *n* denote the number of unique TAD boundaries detected in the chromosome, and let ${D}_i$ denote the average frequency of regulating elements occurring per 10 kb in a 20 kb range centered on *i*th unique TAD boundary.

#### Fold change

The fold change [45] measures the degree of variation in regulatory elements between regions far from and near the TAD boundary. The calculation formula (2) is as follows:


(2) \begin{equation*} \text{Fold}\ \text{change}=\frac{1}{n}\sum\limits_{i=1}^n{\log}_2\left(\frac{A_i}{B_i}\right). \end{equation*}

Let *n* represent the number of unique TAD boundaries detected within the chromosome. ${A}_i$ represents the frequency of biological evidence appearing per 10 kb within the 20 kb range centered on *i*th unique TAD boundary; and ${B}_i$ represents the frequency of biological evidence appearing per 10 kb within a bilateral region between 200 and 500 kb from *i*th unique boundary.

#### Boundary tagged ratio

The boundary tagged ratio [45] is used to describe the degree of enrichment of regulatory elements at the TAD boundary, and the calculation formula (3) is as follows:


(3) \begin{equation*} {\displaystyle \begin{array}{c}\text{Boundary}\ \text{tagged}\ \text{ratio}=\frac{1}{n}\left|S\right|.\end{array}} \end{equation*}

Let *S* be the set of TAD boundaries marked by a biological evidence peak within a 20 kb range upstream and downstream of the boundary. *n* represents the total number of detected TAD boundaries.

#### MoC

To evaluate the similarity between TADs identified by the same caller *via* different normalization methods or bin sizes, we used the measure of concordance (MoC) to compare cluster partitions [46]. The MoC is defined as follows:


(4) \begin{equation*}\kern-.5pc {\displaystyle \begin{array}{@{}l}\text{MoC}\left(\text{P},\text{Q}\right)=\left\{\begin{array}{@{}l}1,\kern7.3pc\text{if}\ {N}_P={N}_Q=1\\{}\frac{1}{\sqrt{N_P{N}_Q-1}}\left(\sum\limits_{i=1}^{N_P}\sum\limits_{j=1}^{N_Q}\frac{{F_{ij}}^2}{P_i{Q}_j}-1\right),\text{otherwise}\end{array}.\right.\ \end{array}} \end{equation*}

In short, *P* and *Q* are the TAD comparison partitions composed of ${N}_P$ and ${N}_Q$, respectively. Each region is defined as a continuous bin interval range. ${P}_i$and ${Q}_j$ are the sizes of two separate TADs in ||${P}_i$|| and ||${Q}_j$||. Finally,||${F}_{ij}$|| is the size of the overlap between two TADs${P}_i$ and ${Q}_j$. The range of the MoC ranges from 0, which represents inconsistent partitions, to 1, which represents identical partitions.

#### TADadj*R*^2^

TADadj*R*^2^ [45] is a measure of the proportion of changes in Hi-C signaling. It can be used to explain differences in contact frequency between TADs over genomic distances and to assess the quality of TAD segmentation structures. Higher TADadj*R*^2^ values indicate that TAD segmentation better reflects significant changes in Hi-C signal patterns that may be associated with a specific biological process or function.

## Results and discussion

In the experiment part, we first evaluated the effectiveness of deepTAD in predicting TAD boundaries at different resolutions. This evaluation focused on analyzing the model's robustness and adaptability to Hi-C contact matrices at different levels of genomic detail.

We then compared the performance of deepTAD with the other five TAD detection methods, including deDoc, TopDom, MSTD, EMTAD, and CATAD. deepTAD was evaluated with these methods on several cell lines (GM12878, K562, IMR90, NHEK, HUVEC, and HMEC) by average peak, fold change, boundary tagged ratio, Moc, and TADadj*R*^2^, which comprehensively assess boundary detection accuracy, structural integrity, and biological relevance.

### Performance of deepTAD across resolutions

We evaluated the performance of deepTAD at different Hi-C resolutions (10, 25, 50, and 100 kb) using all samples from HIC002 for chromosomes 20–22. As shown in Supplementary Table S5, precision is generally lower at higher resolutions, such as 10 kb, due to the dominance of negative samples when predicting the full dataset. While the model was trained with balanced positive and negative samples, the evaluation included all samples, where negative samples are far more abundant. This imbalance naturally increases false positives, leading to lower precision; recall remains relatively high across resolutions, reflecting the model's ability to identify true boundaries. The f1-score, which balances precision and recall, improves at lower resolutions (e.g. 100 kb), suggesting that the model performs more robustly when broader chromatin interaction patterns dominate and noise from finer details is reduced.

Based on these observations, we recommend that users select Hi-C resolutions based on their research goals. Higher resolutions (e.g. 10 kb) are suitable for studying finer TAD structures. However, they may require additional filtering to improve precision, while lower resolutions (e.g. 100 kb) are better for broader chromatin organization patterns.

### Histone enrichment at the TAD boundaries

In this study, we calculate the rates of boundary marking, average peak values, and fold changes in the epigenetic information of the RNA polymerase II, H3K4me3, H3K36me3, H3K9me3, CTCF, RAD21, TSS, SINE, and HK genes, which indicate the level and extent of enrichment or depletion of regulatory elements near the TAD boundary.

#### Average peak

**Figure 2:**
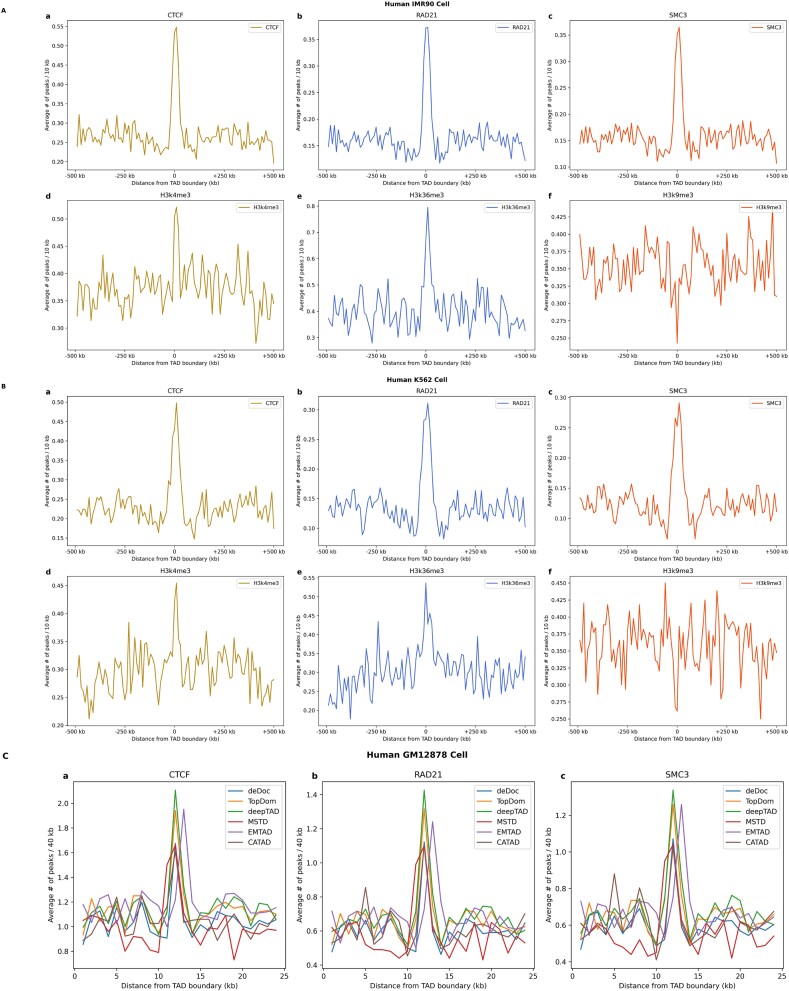
Histone modifications surrounding the boundary. (A) Enrichment analysis of the structural proteins CTCF, RAD21, SMC3, H3k4me3, H3k36me3, and H3k9me3 around the boundaries of TADs identified by deepTAD on chr1 of HIC056 at 25 kb resolution in the IMR90 cell line. (B) Enrichment analysis of the structural proteins CTCF, RAD21, SMC3, H3k4me3, H3k36me3, and H3k9me3 around the boundaries of TADs identified by deepTAD on chr2 of HIC074 at 25 kb resolution in the K562 cell line. (C) Comparison of different methods for transcription factor enrichment at the TAD boundary of chromosome 20 and histone modification in the TAD region in the HIC002 dataset of the GM12878 cell line at 25 kb resolution.

The TAD boundary region is enriched with functional regulatory elements crucial for TAD formation and gene regulation. Key factors include the chromatin insulator CTCF [8] and Cohesin proteins like RAD21 and SMC3 [46], whose binding sites cluster near TAD boundaries (Fig. 2A and B). Additionally, promoter-associated factors such as RNA polymerase II and specific histone modifications (H3K4me3, H3K36me3) [46] are abundant, indicating a link to transcriptional activity (Fig. 2A and B). Conversely, the non-promoter histone modifier H3K9me3 [3], is reduced at TAD boundaries, suggesting a distinct chromatin state associated with gene silencing. Enrichment of transcription start sites and regulatory genes near TAD boundaries highlights their role in gene expression and spatial organization. These findings underscore the importance of TADs in genome structure and regulation, emphasizing the need for further biological insights from TAD boundaries to enhance deepTAD analysis.

As shown in Table 1, the deepTAD method exhibited higher average peaks than the other methods for all seven types of biological evidence, whereas MSTD and EMTAD were superior for only one type of biological evidence. The average peaks of the four regulatory elements measured in the IMR90 cell line were greater than those measured *via* the other methods. The average peaks of the five regulatory elements measured in the K562 cell line were also greater. In addition, we also measured the average peaks of the three cell lines at 50 kb resolution, and the results revealed that deepTAD outperformed the other methods in all tests (Supplementary Tables S6–S10).

**Table 1:** The average peak around the TAD boundaries for nine related biological pieces of evidence using KR-normalized Hi-C data on chr20–22 of HIC002 at 25 kb resolution in the GM12878 cell line

	CTCF	H3K4me3	H3K36me3	HK genes	PolII	RAD21	SINE	TSS	H3K9me3
deDoc	0.434	0.554	0.541	0.105	0.928	0.28	5.513	0.499	0.575
MSTD	0.402	0.534	0.58	0.118	0.78	0.299	5.356	**0.589**	0.591
TopDom	0.458	0.616	0.637	0.129	1.039	0.309	5.431	0.545	0.527
deepTAD	**0.497**	**0.688**	**0.773**	**0.146**	**1.095**	**0.328**	**5.704**	0.585	0.561
CATAD	0.43	0.473	0.377	0.087	0.919	0.283	5.304	0.451	0.547
EMTAD	0.29	0.444	0.616	0.117	0.926	0.143	5.644	0.464	**0.669**

Note: Numbers in bold represent the best result in each column

In this study, we chose KR-normalized Hi-C data on chr20 of HIC002 at 25 kb resolution from the GM12878 cell line to compare different callers (deDoc, TopDom, MSTD, EMTAD, and CATAD) in terms of transcription factor enrichment at the TAD boundary and histone modifications (CTCF, RAD21, and SMC3) in the TAD region. The enrichment or depletion of various regulatory elements near the TAD boundary for all methods was calculated per 40 kb distance from the 500 kb upstream region to the 500 kb downstream region of the TAD boundary. By analyzing these methods in detail, we can obtain information on the details and differences between transcription factor enrichment at TAD boundaries and histone modifications in TAD regions (Fig. 2C). As shown in the figure, deepTAD detected the highest average peaks of proteins at the boundaries, indicating that deepTAD is more effective than other methods in recognizing these features.

#### Fold change

According to Table 2, deepTAD revealed the largest fold change with respect to the six regulatory elements, whereas TopDom, deDoc, and CATAD had the highest magnitude of fold change on one regulatory element. At both 25 and 50 kb resolution, the fold change in deepTAD was the greatest among the four regulatory elements measured by IMR90 (Supplementary Tables S12 and S13). With respect to the five regulatory elements (CTCF, HK genes, RAD21, SMC3, and TSS) measured in the K562 cell line at 25 kb resolution, deepTAD demonstrated the greatest fold change in three regulatory elements, CTCF, RAD21, and SMC3. The fold change in HK genes and TSSs was second only to that of the best-performing caller (Supplementary Table S12). In addition, we measured folding changes in three cell lines, GM12878, K562, and IMR90, at 50 kb resolution, all of which achieved significant results (Supplementary Tables S11, S13, and S14).

**Table 2:** The fold change around the TAD boundaries for nine related biological pieces of evidence using KR-normalized Hi-C data on chr20–22 of HIC002 at 25 kb resolution in the GM12878 cell line

	CTCF	H3K4me3	H3K36me3	HK genes	PolII	RAD21	SINE	TSS	H3K9me3
deDoc	0.776	0.542	0.145	0.127	0.301	0.912	**0.029**	0.563	−0.173
MSTD	0.452	0.376	0.213	0.361	−0.073	0.759	0.022	0.537	−0.211
TopDom	0.795	0.624	0.373	0.279	**0.365**	1.005	−0.051	0.641	−0.191
deepTAD	**0.855**	**0.643**	**0.524**	**0.41**	0.319	**1.063**	0.005	**0.688**	−0.205
CATAD	0.809	0.051	−0.26	−0.144	0.164	0.943	−0.037	0.384	**−0.219**
EMTAD	0.036	0.035	0.286	0.211	0.191	−0.159	0.024	0.134	0.091

Note: Numbers in bold represent the best result in each column

#### Boundary tagged ratio

As shown in Table 3, deepTAD had the highest rate of boundary labeling on the seven regulatory elements, whereas deDoc had the highest boundary tagged ratio on the SINE and H3K9me3. Regulatory elements measured in the IMR90 and K562 cell lines at 25 and 50 kb resolutions revealed that deepTAD achieved a significant boundary tagged ratio (Supplementary Tables S16–S20).

**Table 3:** Boundary tagged ratio around the TAD boundaries (25 kb) for nine related biological pieces of evidence *via* KR-normalized Hi-C data on chr20–22 of HIC002 at 25 kb resolution in the GM12878 cell line

	CTCF	H3K4me3	H3K36me3	HK genes	PolII	RAD21	SINE	TSS	H3K9me3
deDoc	0.756	0.449	0.406	0.189	0.734	0.648	**0.989**	0.542	**0.696**
MSTD	0.771	0.405	0.379	0.185	0.718	0.674	0.969	0.529	0.687
TopDom	0.795	0.484	0.441	0.207	0.74	0.72	0.982	0.585	0.683
deepTAD	**0.832**	**0.524**	**0.486**	**0.246**	**0.737**	**0.777**	0.977	**0.609**	0.694
CATAD	0.734	0.379	0.323	0.156	0.677	0.638	0.979	0.496	0.695
EMTAD	0.636	0.363	0.376	0.166	0.682	0.492	0.981	0.52	0.693

Note: Numbers in bold represent the best result in each column

From the above analysis, we observe that the boundaries detected by deepTAD exhibit higher average peak values, greater fold changes, and improved boundary tagged ratio. These metrics indicate a stronger correlation between deepTAD predicted boundaries and specific histone modifications, this robust correlation underscores deepTAD’s capability to capture biologically meaningful TAD boundaries. By leveraging this strength, deepTAD facilitates a more comprehensive understanding of gene regulatory mechanisms and chromatin organization.

### TAD identification with different resolution and normalization methods

**Figure 3:**
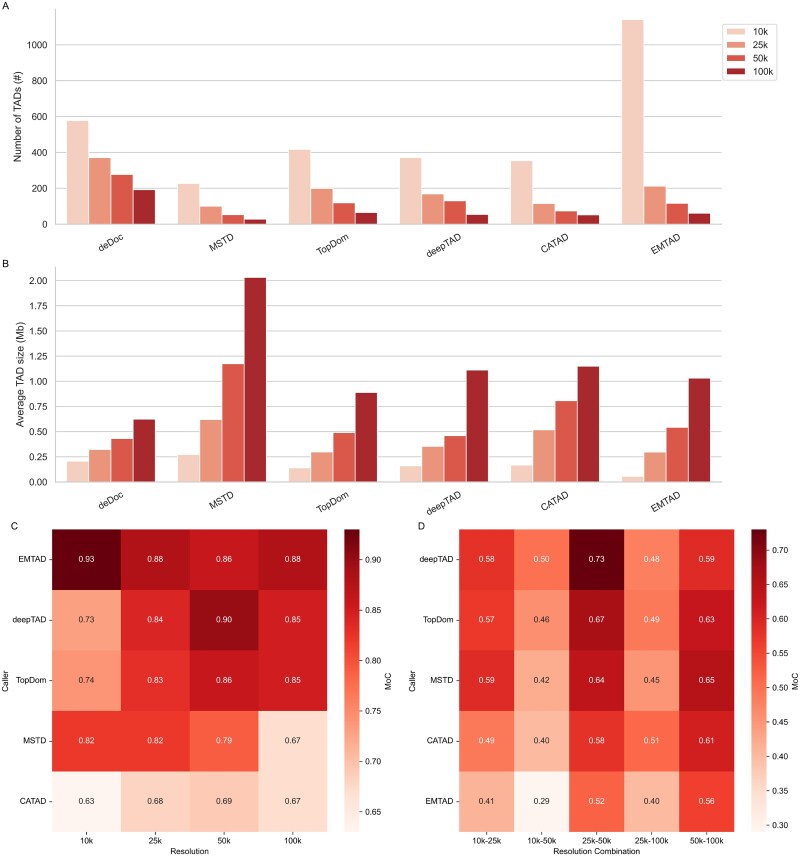
Identification of TADs from different TAD callers at various resolutions (10, 25, 50, and 100 kb). (A) Number of TADs recognized on chr20 per caller at different resolutions. (B) The average size of TADs recognized on chr20 per caller at different resolutions. (C) Using the MoC to evaluate the consistency between the TAD partitions of each TAD caller obtained from the normalized contact matrix of the KR and VC at four different resolutions (10, 25, 50, and 100 kb). (D) Consistency between TAD partitions obtained at different resolutions was assessed *via* MoC in pairs (10 kb versus 50 kb, 10 kb versus 100 kb, etc.; the results of KR normalization data are displayed here).

To validate the effectiveness of the proposed method, we generated Hi-C contact matrices for the GM12878, IMR90, K562, NHEK, HMEC, and HUVEC lines at different resolutions (10, 25, 50, and 100 kb) on chromosomes 20–22 *via* two popular normalization methods, namely, KR and VC. First, we examined the number of TADs and their average size obtained by each TAD caller at different resolutions on chr20 of HIC002 *via* the KR normalization method (Fig. 3A and B). We found that the average TAD size usually increases with bin size, whereas the number of TADs decreases with bin size. At 10 kb resolution, EMTAD recognized the largest number of TADs and, accordingly, the smallest average size of recognized TADs. In addition, the MSTD method had the largest average size at 100 kb resolution, whereas the deepTAD method yielded TADs with an average size comparable to those of other TAD recognition methods.

As shown in Table 4 and Supplementary Fig. S1, TAD on chr20 was detected in different cell lines (IMR90, K562, NHEK, HMEC, and HUVEC) at 25 kb resolution. MSTD identified the fewest TADs with the largest average size, while TopDom detected more TADs with a smaller average size. deDoc, designed to focus on nested TAD structures, detected the highest number of TADs, primarily smaller ones (<200 kb). CATAD and deDoc recognized significantly more TADs in the IMR90, HMEC, and NHEK cell lines. The number of TADs detected by deepTAD and the other TAD callers remained stable. Size distribution analysis revealed that deepTAD predominantly identified TADs in the 200–500 kb range, aligning with the sizes commonly reported in biological studies. By contrast, MSTD and CATAD were biased toward larger TADs (>500 kb). Among the cell lines, the IMR90 cell line had a greater number of TADs, but its average TAD size was smaller.

**Table 4:** The number and average size of TADs detected on chr20 of different cell lines with 25 kb resolution and KR normalization

Cell		CATAD	EMTAD	MSTD	TopDom	deDoc	deepTAD
HMEC	#TAD	113	201	103	216	368	131
	Av.size (Mb)	0.527	0.313	0.604	0.27	0.324	0.455
HUVEC	#TAD	86	210	97	184	238	176
	Av.size (Mb)	0.694	0.3	0.64	0.32	0.502	0.338
IMR90	#TAD	110	199	96	181	367	161
	Av.size (Mb)	0.542	0.316	0.645	0.323	0.32	0.37
K562	#TAD	76	197	94	192	245	180
	Av.size (Mb)	0.787	0.32	0.659	0.308	0.487	0.331
NHEK	#TAD	122	208	109	214	344	165
	Av.size (Mb)	0.489	0.303	0.573	0.274	0.347	0.361

**Figure 4:**
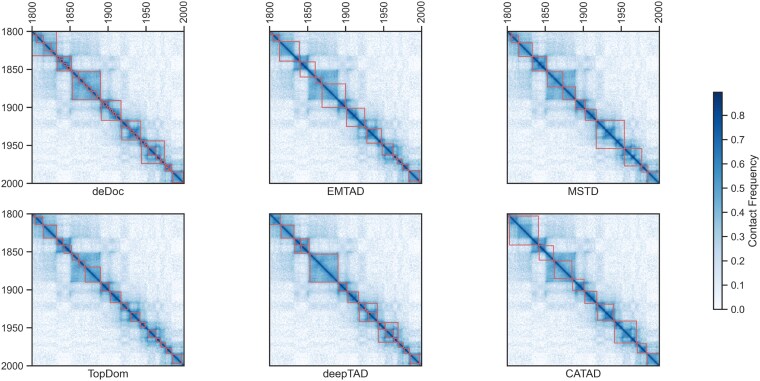
Heatmap and annotated TADs of different callers found on chromosome 20 (1800–2000) at 25 kb resolution *via* KR-normalized HIC002 samples from the human GM12878 cell line.

For the five methods compared, only deDoc could identify nested TAD, and we counted the number of nests of deepTAD and deDoc on chromosome 20 in five cell lines and the results are shown in Supplementary Table S21. According to the data analysis in Fig. 4 and Supplementary Table S21, deDoc and deepTAD show significant differences in their approaches to TAD segmentation. deDoc demonstrates a higher resolution capability, identifying a greater number of smaller TADs. It even attempts fine-grained segmentation in regions with weaker signals, highlighting its ability to capture local details. deepTAD, on the other hand, favors the identification of larger and smoother TADs, especially in regions with stronger signals. Its segmentation results emphasize the stability and coherence of the global structure, resulting in fewer but more robustly defined TADs.

We quantified the consistency between TAD partitions obtained using KR and VC normalization methods for each TAD caller across resolutions. Except for CATAD, other callers showed high consistency in TAD identification across normalizations (MoC > 0.8) (Fig. 3C). Despite different normalization methods, deepTAD maintained high partition consistency at 25 kb, 50 kb, and 100 kb resolutions, indicating its robustness in TAD partitioning regardless of normalization and resolution. We also assessed deepTAD's stability in boundary identification across resolutions, finding high consistency (73%) at finer resolutions (25 kb versus 50 kb) and moderate consistency (59%) at medium resolutions (100 kb versus 50 kb) (Fig. 3D). Even at finer resolutions (10 kb versus 50 kb), 50% of boundaries remained consistent. Among TAD callers, deepTAD achieved the highest MoC, particularly in IMR90, HMEC, and HUVEC cell lines at resolutions like 10 kb versus 25 kb. In the NHEK and K562 lines, it performed best at 25 kb versus 50 kb, though slightly lower at other resolutions (Supplementary Fig. S2).

### Performance of TAD identification

Next, we use TADadj*R*^2^ to evaluate high degree of Hi-C signal variance.

**Figure 5:**
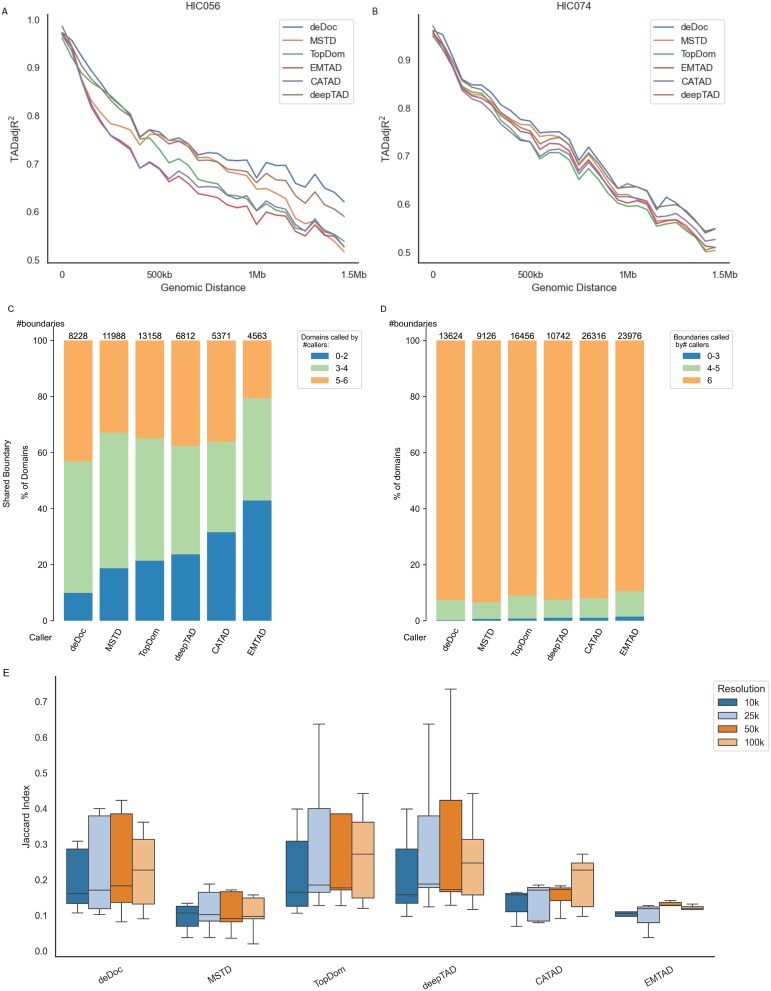
Assessment of different methods using TADadjR2, boundary conservation, and Jaccard Index (JI). Performance of proportions of Hi-C signal variability explained by the TADs (measured by TADadj*R*^2^) between a pair of loci across genomic distances (0–1.5 M) for different TAD callers on KR normalization Hi-C data. (A) on chr1-X of HIC056 at 25 kb resolution in the IMR90 cell line. (B) On chr1-X of HIC074 at 25 kb resolution in the K562 cell line. (C) Conservation percentage of the TAD domain in HIC056 samples from the IMR90 cell line across the 6 TAD callers at 25 kb resolution. (D) Conservation percentage of TAD boundaries on HIC056 samples of IMR90 cell lines across the 6 TAD callers at 25 kb resolution. (E) Similarity (measured by JI) of TAD boundaries between a TAD caller and the others on chr20 of HIC002 in GM12878 cell lines at different resolutions.

Over almost the entire range of genomic distances (0–1.5 Mb), we measure the TADadj*R*^2^ values of different methods *via* KR-normalized Hi-C data from chr20–22 of HIC002 at 25 kb resolution for the GM12878 cell line (Table 5). The results showed that the deepTAD achieved higher TADadj*R*^2^ values on chr20 compared to other methods, whereas the TADadj*R*^2^ values on chr21 and chr22 were slightly lower than those of the best caller. The TADadj*R*^2^ values of deepTAD were slightly lower than those of deDoc for the HIC056 sample from IMR90 and the HIC074 sample from the K562 cell line (Fig. 5A and B). Overall, the TADadj*R*^2^ values of deepTAD were slightly lower than those of the individual methods for some chromosomes and samples. deepTAD demonstrated a better ability to discriminate between the TAD and non-TAD regions. This performance suggests that deepTAD can effectively identify and distinguish significant signal changes in Hi-C data with good adaptability and stability and performs well across multiple datasets and resolutions.

**Table 5:** Performance of the proportions of Hi-C signal variability explained by the TADs (measured by TADadj*R*^2^) between a pair of loci across genomic distances (0–1.5 M), at a 25 kb resolution on GM12878 cells for HIC002 on chromosomes 20–22

	deDoc	MSTD	TopDom	EMTAD	CATAD	deepTAD
HIC002_chr20	0.771	0.747	0.725	0.725	0.716	0.773
HIC002_chr21	0.696	0.63	0.591	0.666	0.595	0.672
HIC002_chr22	0.675	0.655	0.563	0.693	0.589	0.645

Differences in the number, size, and spacing of TADs reveal many preferences and perspectives among TAD callers. Such differences are common in practice, as different tools interpret the data based on their algorithms and parameters. Various TAD callers may focus more on different aspects, e.g. some callers are more inclined to identify larger TADs. In contrast, others focus more on smaller TADs, which leads to differences in the number and size of TADs. However, TAD boundary conservatism and TAD domain conservatism are two other important aspects of understanding the differences between TAD callers. In some cases, different TAD callers may reach a certain degree of conservatism in TAD boundaries (Fig. 5C and D), deepTAD recognizes >40% of the boundaries identified by the other five callers, and >80% of the TAD domains are also identified by the other five callers. In addition, even for the same dataset, the low similarity of TAD boundaries between different TAD callers (Fig. 5E) reflects differences in their understanding and definition of TAD boundaries. Although some callers may agree on some TADs, overall, there is a lack of consensus among different TAD callers on the same data. To demonstrate this difference more clearly, Fig. 4 shows a heatmap of a portion of the Hi-C contact matrix and labels the TADs recognized by each TAD caller with a red block. This graphic demonstrates the differences between the different callers and reveals their varying interpretations of the number, size, and boundaries of the TADs. This diversity and variability have allowed researchers to study the structure of chromosomes from different perspectives and gain a more complete understanding.

In order to verify the feature extraction ability and the robustness of the original model, we added different levels of Gaussian noise to the Hi-C contact matrix. By adjusting the noise ratio, we explored the impact of data quality variations on model performance, and the relevant experimental results are shown in Supplementary Table S22. The results show that introducing Gaussian noise at different levels does not significantly affect the model's ability to extract features, as evidenced by the stable performance across all metrics. This suggests that the model is robust to small variations in data quality, ensuring reliable performance under different noise conditions.

In addition to using cosine similarity, we also evaluated the performance of Euclidean distance for detecting nested TADs. Specifically, we applied both methods to analyze nested TADs on chromosomes 20–22 using HIC002 data and evaluated their segmentation ability using the TADadj*R*^2^ metric. The experimental results (Supplementary Table S23) show that cosine similarity outperforms Euclidean distance in terms of the TADadj*R*^2^ score. This suggests that cosine similarity is more effective at capturing the relative pattern similarities between regions, likely due to its focus on angular relationships rather than absolute distances. Based on these findings, we chose cosine similarity to detect nested TADs as it better reflects the structural characteristics of the genomic regions under analysis.

## Conclusion

Our current understanding of chromatin structure has been enhanced by the discovery of TADs, which also opens new possibilities for epigenetic studies and cancer etiology research. Observing how TADs form and regulate genes, we can better understand how gene spatial layout and epigenetic modifications are interrelated. In this work, we propose a new TAD identification method, called deepTAD, based on a CNN-transformer model for feature extraction from the Hi-C contact matrix. This method predicts the boundaries in the matrix of new samples by training the model, screens out the false-positive boundaries *via* the Wilcoxon rank-sum test, and finally completes the TAD assembly *via* cosine similarity. A comparison of deepTAD with five other methods was conducted to ensure validity and accuracy. The results of these experiments show that deepTAD can detect TADs efficiently and accurately. This method performs well with respect to the TAD domain and boundary conservatism. Additionally, deepTAD showed advantages in terms of accuracy and superiority in histone modification mark enrichment analysis experiments.

**Key Points**

- The identification of topologically associated domains helps scientists gain a deeper understanding of the 3D structure of the genome and its functional relationships and can provide new perspectives for disease research and genomics studies.
- Based on Hi-C data, a deep learning and advanced feature fusion approach to recognize TAD is proposed. The method is described in detail with the help of images and formulas.
- In order to evaluate the performance of deepTAD and the other five TAD-identifying methods, we analyzed some relevant computational experiments in six datasets.

## Supplementary Material

Supplementray_Materials_bbaf127

## Data Availability

The Hi-C dataset used in this study was obtained from the Gene Expression Omnibus (GEO) database under accession number GSE63525. The accession details for the ChIP-Seq data are provided in Supplementary Table S2.
