# Supplementary material for: deepTAD: an approach for identifying topologically associated domains based on convolutional neural network and transformer model
Source: Brief Bioinform. 2025 Mar 25;26(2):bbaf127. doi: 10.1093/bib/bbaf127 (PMC11934553; doi:10.1093/bib/bbaf127)
Supplement: Supplementray_Materials_bbaf127 [file supplementray_materials_bbaf127.docx]

**Supplementary Figure S1.** TAD size distribution in different cell lines.


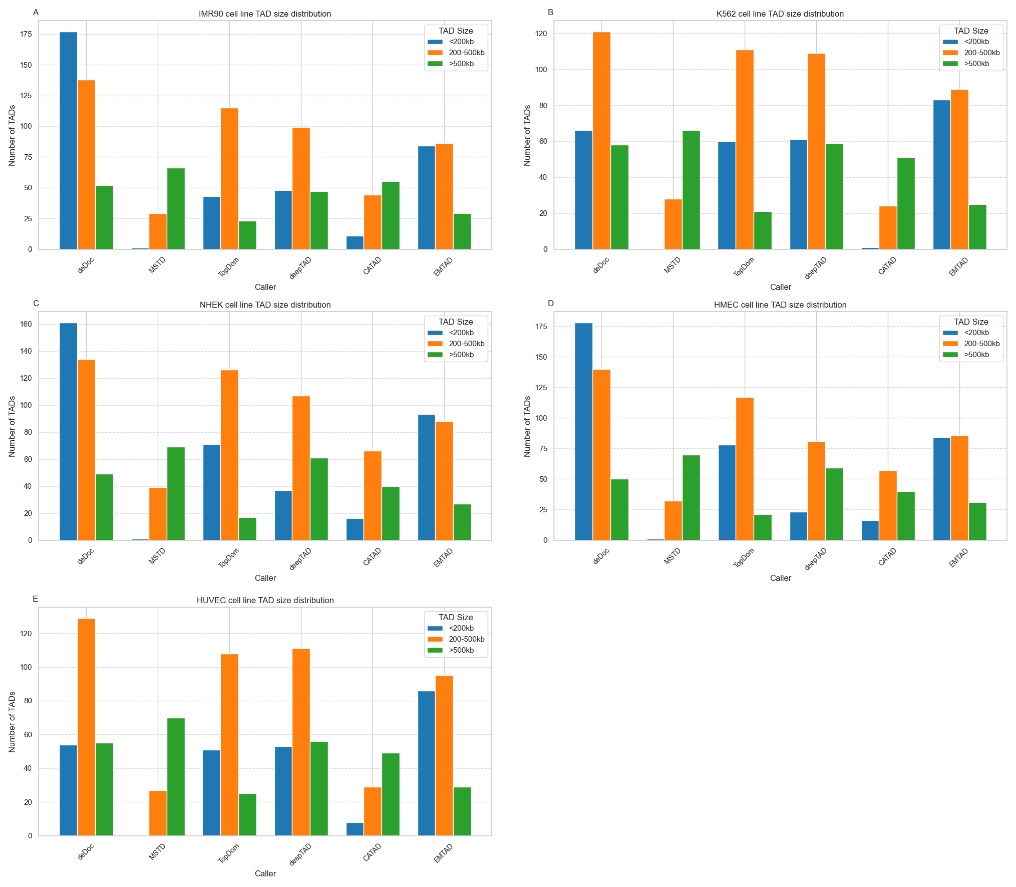


**Supplementary Figure S2**. MoC values for different cell lines.


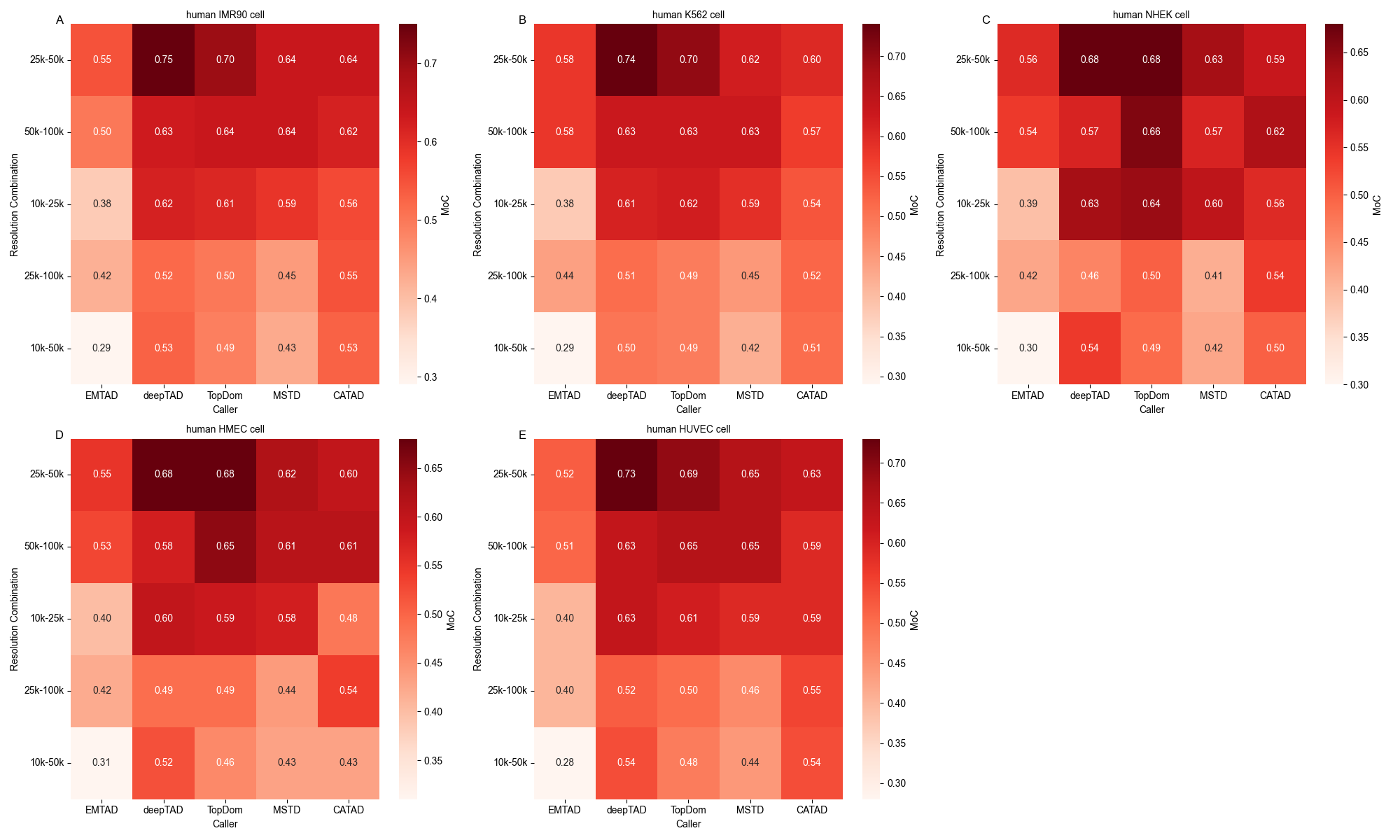


**Supplementary Table S1**. Summary of Hi-C datasets used in this study.

| Datasets | Brief description | Data source |
| --- | --- | --- |
| Experimental dataset  Rao *et al.* 2014 | This dataset contains 200 samples in total. The 29 replicate samples in human GM12878 cell line are HIC001-HIC029.  The 7 replicate samples in human IMR90 cell line are HIC050-HIC056.  The 6 replicate samples in human HMEC cell line are HIC058-HIC063.  The 6 replicate samples in human K562 cell line are HIC069-HIC074.  The replicate samples in IMR90 cell line used for analysis of biological and technical replicate is HIC056.  The replicate samples in K562 cell line used for analysis of replicate with similar sequencing depth is HIC074.  The replicate samples in NHEK cell line used for analysis of replicates are HIC065-HIC067.  The replicate samples in HUVEC cell line used for analysis of replicates are HIC080-HIC082. | GEO accession ID: GSE63525 |

**Supplementary** **Table S2.** Summary of download information of ChIP-seq peaks, TSSs, SINEs and housekeeping genes.

| Species | Cell lines | Name | Download information |
| --- | --- | --- | --- |
| Human | GM12878 | CTCF | http://hgdownload.cse.ucsc.edu/goldenpath/hg19/encodeDCC/wgEncodeAwgTfbsUniform/wgEncodeAwgTfbsSydhGm12878Ctcfsc15914c20UniPk.narrowPeak.gz |
|  |  | RAD21 | http://hgdownload.cse.ucsc.edu/goldenpath/hg19/encodeDCC/wgEncodeAwgTfbsUniform/wgEncodeAwgTfbsSydhGm12878Rad21IggrabUniPk.narrowPeak.gz |
|  |  | SMC3 | http://hgdownload.cse.ucsc.edu/goldenpath/hg19/encodeDCC/wgEncodeAwgTfbsUniform/wgEncodeAwgTfbsSydhGm12878Smc3ab9263IggmusUniPk.narrowPeak.gz |
|  |  | H3K4me3 | http://hgdownload.cse.ucsc.edu/goldenpath/hg19/encodeDCC/wgEncodeUwHistone/wgEncodeUwHistoneGm12878H3k4me3StdPkRep1.narrowPeak.gz |
|  |  | H3K36me3 | http://hgdownload.cse.ucsc.edu/goldenpath/hg19/encodeDCC/wgEncodeUwHistone/wgEncodeUwHistoneGm12878H3k36me3StdPkRep1.narrowPeak.gz |
|  |  | H3K9me3 | http://hgdownload.cse.ucsc.edu/goldenpath/hg19/encodeDCC/wgEncodeBroadHistone/wgEncodeBroadHistoneGm12878H3k9me3StdPk.broadPeak.gz |
|  |  | Polymerase II | http://hgdownload.cse.ucsc.edu/goldenpath/hg19/encodeDCC/wgEncodeOpenChromChip/wgEncodeOpenChromChipGm12878Pol2Pk.narrowPeak.gz |
|  |  | SINEs | http://hgdownload.soe.ucsc.edu/goldenPath/hg19/database/rmsk.txt.gz |
|  | shared | TSSs | retrievable from mysql.cse.ucsc.edu |
|  |  | Housekeeping genes | http://www.tau.ac.il/~elieis/HKG/HK_genes.txt |
|  | IMR90 | CTCF | http://hgdownload.cse.ucsc.edu/goldenpath/hg19/encodeDCC/wgEncodeAwgTfbsUniform/wgEncodeAwgTfbsSydhImr90CtcfbIggrabUniPk.narrowPeak.gz |
|  |  | RAD21 | http://hgdownload.cse.ucsc.edu/goldenpath/hg19/encodeDCC/wgEncodeAwgTfbsUniform/wgEncodeAwgTfbsSydhImr90Rad21IggrabUniPk.narrowPeak.gz |
|  | K562 | CTCF | http://hgdownload.cse.ucsc.edu/goldenpath/hg19/encodeDCC/wgEncodeAwgTfbsUniform/wgEncodeAwgTfbsSydhK562CtcfbIggrabUniPk.narrowPeak.gz |
|  |  | RAD21 | http://hgdownload.cse.ucsc.edu/goldenpath/hg19/encodeDCC/wgEncodeAwgTfbsUniform/wgEncodeAwgTfbsSydhK562Rad21UniPk.narrowPeak.gz |
|  |  | SMC3 | http://hgdownload.cse.ucsc.edu/goldenpath/hg19/encodeDCC/wgEncodeAwgTfbsUniform/wgEncodeAwgTfbsSydhK562Smc3ab9263IggrabUniPk.narrowPeak.gz |

**Supplementary Table S3.** Impact of key hyperparameters (learning rate, kernel size, number of transformer heads) on model performance.

|  |  |  | precision | recall | f1-score |
| --- | --- | --- | --- | --- | --- |
| seed=123 | learning rate | 0.001 | 0.903±0.007 | 0.895±0.006 | 0.899±0.001 |
|  |  | 0.0001 | 0.9±0.009 | 0.9±0.008 | 0.9±0.002 |
|  |  | 0.0003 | 0.896±0.007 | 0.908±0.007 | 0.902±0.001 |
| seed=123,  lr=0.0003 | kernel_size | (128,5*5)(64,5*5) | 0.906±0.007 | 0.896±0.007 | 0.901±0.001 |
|  |  | (128,5*5)(64,3*3) | 0.904±0.009 | 0.897±0.011 | 0.9±0.001 |
|  |  | (128,3*3)(64,5*5) | 0.9±0.001 | 0.898±0.013 | 0.903±0.012 |
|  |  | (128,3*3)(64,3*3) | 0.896±0.007 | 0.908±0.007 | 0.902±0.001 |
| seed=123, lr=0.0003  kernel_size=(128,3*3)(64,3*3) | number of transformer heads | 2 | 0.884±0.035 | 0.896±0.006 | 0.9±0.001 |
|  |  | 4 | 0.896±0.007 | 0.908±0.007 | 0.902±0.001 |

**Supplementary Table S4.** Impact of key hyperparameters (random seeds) on model performance.

| seed | precision | recall | f1-score |
| --- | --- | --- | --- |
| 1 | 0.904±0.008 | 0.898±0.007 | 0.901±0.0 |
| 42 | 0.911±0.007 | 0.891±0.007 | 0.901±0.01 |
| 123 | 0.908±0.01 | 0.895±0.01 | 0.901±0.01 |
| 999 | 0.896±0.007 | 0.908±0.007 | 0.902±0.001 |
| 2024 | 0.9±0.01 | 0.901±0.01 | 0.9±0.0 |

**Supplementary Table S5.** Model performance at different resolutions for predicting HIC002 chromosomes 20–22.

| resolution |  | precision | recall | f1-score |
| --- | --- | --- | --- | --- |
| 10kb | HIC002_chr20 | 0.271 | 0.763 | 0.4 |
|  | HIC002_chr21 | 0.243 | 0.842 | 0.377 |
|  | HIC002_chr22 | 0.32 | 0.778 | 0.453 |
| 25kb | HIC002_chr20 | 0.547 | 0.834 | 0.66 |
|  | HIC002_chr21 | 0.507 | 0.752 | 0.606 |
|  | HIC002_chr22 | 0.565 | 0.867 | 0.684 |
| 50kb | HIC002_chr20 | 0.566 | 0.862 | 0.683 |
|  | HIC002_chr21 | 0.59 | 0.676 | 0.63 |
|  | HIC002_chr22 | 0.644 | 0.817 | 0.72 |
| 100kb | HIC002_chr20 | 0.633 | 0.803 | 0.708 |
|  | HIC002_chr21 | 0.583 | 0.8 | 0.674 |
|  | HIC002_chr22 | 0.654 | 0.766 | 0.706 |

**Supplementary** **Table S6.** The average peak around the TAD boundaries for nine related biological pieces of evidence using KR-normalized Hi-C data on chr20-22 of HIC002 at 50 kb resolution in the GM12878 cell line.

|  | CTCF | H3K4me3 | H3K36me3 | HK genes | PolII | RAD21 | SINE | TSS | H3K9me3 |
| --- | --- | --- | --- | --- | --- | --- | --- | --- | --- |
| deDoc | 0.369 | 0.465 | 0.606 | 0.114 | 0.959 | 0.249 | 5.528 | 0.467 | 0.528 |
| MSTD | 0.329 | 0.426 | 0.579 | 0.098 | **1.401** | 0.154 | 5.618 | **0.823** | 0.438 |
| TopDom | **0.447** | 0.543 | 0.648 | 0.113 | 1.106 | 0.31 | 5.832 | 0.541 | 0.433 |
| deepTAD | 0.438 | **0.616** | **0.711** | **0.114** | 1.074 | **0.292** | 5.944 | 0.537 | 0.44 |
| CATAD | 0.403 | 0.416 | 0.572 | 0.098 | 0.869 | 0.249 | 5.615 | 0.554 | 0.446 |
| EMTAD | 0.276 | 0.579 | 0.409 | 0.115 | 0.964 | 0.146 | **6.016** | 0.494 | **0.635** |

**Supplementary** **Table S7.** The average peak around the TAD boundaries for four related biological pieces of evidence using KR-normalized Hi-C data on chr1-X of HIC056 at 50kb resolution in the IMR90 cell line.

|  | CTCF | HK genes | RAD21 | TSS |
| --- | --- | --- | --- | --- |
| deDoc | 0.351 | 0.113 | 0.294 | 0.45 |
| MSTD | 0.28 | 0.11 | 0.223 | 0.412 |
| TopDom | 0.406 | 0.141 | 0.333 | 0.548 |
| deepTAD | **0.444** | **0.152** | **0.368** | **0.576** |
| CATAD | 0.383 | 0.118 | 0.317 | 0.481 |
| EMTAD | 0.189 | 0.096 | 0.149 | 0.348 |

**Supplementary** **Table S8.** The average peak around the TAD boundaries for five related biological pieces of evidence using KR-normalized Hi-C data on chr1-X of HIC074 at 50kb resolution in the K562 cell line.

|  | CTCF | HK genes | RAD21 | SMC3 | TSS |
| --- | --- | --- | --- | --- | --- |
| deDoc | 0.371 | 0.096 | 0.174 | 0.208 | 0.413 |
| MSTD | 0.323 | 0.091 | 0.132 | 0.163 | 0.394 |
| TopDom | 0.44 | 0.123 | 0.203 | 0.242 | 0.454 |
| deepTAD | **0.456** | **0.125** | **0.211** | **0.258** | 0.497 |
| CATAD | 0.426 | 0.106 | 0.182 | 0.233 | **0.516** |
| EMTAD | 0.21 | 0.087 | 0.058 | 0.087 | 0.312 |

**Supplementary** **Table S9.** The average peak around the TAD boundaries for five related biological pieces of evidence using KR-normalized Hi-C data on chr1-X of HIC074 at 25kb resolution in the K562 cell line.

|  | CTCF | HK genes | RAD21 | SMC3 | TSS |
| --- | --- | --- | --- | --- | --- |
| deDoc | 0.411 | 0.093 | 0.195 | 0.235 | 0.461 |
| MSTD | 0.375 | 0.094 | 0.165 | 0.202 | 0.43 |
| TopDom | 0.454 | 0.116 | 0.212 | 0.264 | 0.463 |
| deepTAD | **0.527** | **0.126** | **0.252** | **0.307** | **0.553** |
| CATAD | 0.453 | 0.103 | 0.216 | 0.262 | 0.544 |
| EMTAD | 0.206 | 0.072 | 0.06 | 0.088 | 0.271 |

**Supplementary** **Table S10.** The average peak around the TAD boundaries for four related biological pieces of evidence using KR-normalized Hi-C data on chr1-X of HIC056 at 25kb resolution in the IMR90 cell line.

|  | CTCF | HK genes | RAD21 | TSS |
| --- | --- | --- | --- | --- |
| deDoc | 0.409 | 0.112 | 0.352 | 0.487 |
| MSTD | 0.329 | 0.115 | 0.257 | 0.504 |
| TopDom | 0.476 | 0.14 | 0.4 | 0.58 |
| deepTAD | **0.544** | **0.165** | **0.462** | **0.641** |
| CATAD | 0.425 | 0.11 | 0.355 | 0.487 |
| EMTAD | 0.192 | 0.09 | 0.154 | 0.327 |

**Supplementary Table 11.** The fold change around the TAD boundaries for nine related biological evidence using KR-normalized Hi-C data on chr20-22 of HIC002 at 50 kb resolution in the GM12878 cell line.

|  | CTCF | H3K4me3 | H3K36me3 | HK genes | PolII | RAD21 | SINE | TSS | H3K9me3 |
| --- | --- | --- | --- | --- | --- | --- | --- | --- | --- |
| deDoc | 0.52 | 0.308 | 0.352 | 0.203 | 0.259 | 0.753 | 0.021 | 0.352 | -0.23 |
| MSTD | 0.202 | -0.096 | -0.038 | 0.03 | **0.598** | -0.019 | 0.065 | **1.044** | **-0.654** |
| TopDom | 0.687 | 0.413 | 0.445 | **0.174** | 0.405 | **0.888** | 0.07 | 0.591 | -0.606 |
| deepTAD | **0.713** | **0.51** | **0.542** | 0.173 | 0.375 | 0.856 | **0.09** | 0.505 | -0.607 |
| CATAD | 0.532 | -0.275 | -0.281 | -0.175 | -0.176 | 0.629 | -0.081 | 0.228 | -0.57 |
| EMTAD | -0.12 | 0.142 | -0.522 | 0.083 | -0.018 | -0.279 | 0.06 | 0.212 | 0.019 |

**Supplementary** **Table S12.** The fold change around the TAD boundaries for four related biological pieces of evidence using KR-normalized Hi-C data on chr1-X of HIC056 at 25kb resolution in the IMR90 cell line.

|  | CTCF | HK genes | RAD21 | TSS |
| --- | --- | --- | --- | --- |
| deDoc | 1.019 | 0.384 | 1.104 | 0.621 |
| MSTD | 0.869 | 0.601 | 0.825 | 0.716 |
| TopDom | 1.163 | 0.595 | 1.216 | 0.784 |
| deepTAD | **1.274** | **0.682** | **1.326** | **0.84** |
| CATAD | 1.042 | 0.281 | 1.089 | 0.498 |
| EMTAD | -0.135 | 0.095 | -0.183 | 0.063 |

**Supplementary** **Table S13.** The fold change around the TAD boundaries for four related biological pieces of evidence using KR-normalized Hi-C data on chr1-X of HIC056 at 50kb resolution in the IMR90 cell line.

|  | CTCF | HK genes | RAD21 | TSS |
| --- | --- | --- | --- | --- |
| deDoc | 0.814 | 0.393 | 0.865 | 0.529 |
| MSTD | 0.746 | 0.617 | 0.698 | 0.591 |
| TopDom | 0.944 | 0.596 | 0.946 | 0.712 |
| deepTAD | **1** | **0.643** | **1.025** | **0.76** |
| CATAD | 0.853 | 0.301 | 0.858 | 0.481 |
| EMTAD | -0.173 | 0.102 | -0.188 | 0.09 |

**Supplementary** **Table S14.** The fold change around the TAD boundaries for five related biological pieces of evidence using KR-normalized Hi-C data on chr1-X of HIC074 at 50kb resolution in the K562 cell line.

|  | CTCF | HK genes | RAD21 | SMC3 | TSS |
| --- | --- | --- | --- | --- | --- |
| deDoc | 0.745 | 0.255 | 1.326 | 1.063 | 0.424 |
| MSTD | 0.705 | 0.159 | 1.119 | 0.95 | 0.407 |
| TopDom | 0.791 | 0.411 | 1.341 | 1.089 | 0.491 |
| deepTAD | **0.825** | **0.42** | **1.358** | **1.155** | 0.537 |
| CATAD | 0.783 | 0.273 | 1.256 | 1.054 | **0.623** |
| EMTAD | -0.115 | 0.093 | -0.36 | -0.262 | 0.01 |

**Supplementary** **Table S15.** The fold change around the TAD boundaries for five related biological pieces of evidence using KR-normalized Hi-C data on chr1-X of HIC074 at 25kb resolution in the K562 cell line.

|  | CTCF | HK genes | RAD21 | SMC3 | TSS |
| --- | --- | --- | --- | --- | --- |
| deDoc | 0.899 | 0.184 | 1.525 | 1.287 | 0.595 |
| MSTD | 0.776 | 0.314 | 1.284 | 1.078 | 0.446 |
| TopDom | 0.895 | **0.459** | 1.432 | 1.27 | 0.558 |
| deepTAD | **1.017** | 0.451 | **1.606** | **1.402** | 0.727 |
| CATAD | 0.971 | 0.343 | 1.522 | 1.28 | **0.749** |
| EMTAD | -0.061 | 0.036 | -0.194 | -0.137 | 0.003 |

**Supplementary** **Table S16.** Boundary tagged ratio around the TAD boundaries (25 kb) for nine related biological pieces of evidence via KR-normalized Hi-C data on chr20-22 of HIC002 at 50 kb resolution in the GM12878 cell line.

|  | CTCF | H3K4me3 | H3K36me3 | HK genes | PolⅡ | RAD21 | SINE | TSS | H3K9me3 |
| --- | --- | --- | --- | --- | --- | --- | --- | --- | --- |
| deDoc | 0.835 | 0.567 | 0.527 | 0.255 | 0.866 | 0.778 | **0.992** | 0.699 | 0.864 |
| MSTD | 0.853 | 0.534 | 0.491 | 0.241 | 0.853 | 0.784 | 0.974 | 0.707 | 0.767 |
| TopDom | 0.893 | 0.628 | 0.586 | 0.283 | 0.862 | 0.838 | 0.986 | 0.738 | **0.869** |
| deepTAD | **0.91** | **0.652** | **0.595** | 0.294 | **0.878** | **0.875** | 0.986 | **0.767** | 0.849 |
| CATAD | 0.85 | 0.561 | 0.497 | **0.301** | 0.884 | 0.78 | 0.988 | 0.711 | 0.832 |
| EMTAD | 0.835 | 0.597 | 0.535 | 0.288 | 0.872 | 0.786 | 0.975 | 0.712 | 0.877 |

**Supplementary** **Table S17.** Boundary tagged ratio around the TAD boundaries (25 kb) for four related biological pieces of evidence via KR-normalized Hi-C data on chr1-X of HIC056 at 25kb resolution in the IMR90 cell line.

|  | CTCF | HK | RAD21 | TSS |
| --- | --- | --- | --- | --- |
| deDoc | 0.851 | 0.234 | 0.822 | 0.663 |
| MSTD | 0.822 | 0.225 | 0.772 | 0.636 |
| TopDom | 0.925 | 0.277 | 0.902 | 0.73 |
| deepTAD | **0.935** | **0.295** | **0.919** | **0.744** |
| CATAD | 0.859 | 0.248 | 0.827 | 0.687 |
| EMTAD | 0.76 | 0.196 | 0.702 | 0.629 |

**Supplementary** **Table S18.** Boundary tagged ratio around the TAD boundaries (25 kb) for four related biological pieces of evidence via KR-normalized Hi-C data on chr1-X of HIC056 at 50kb resolution in the IMR90 cell line.

|  | CTCF | HK genes | RAD21 | TSS |
| --- | --- | --- | --- | --- |
| deDoc | 0.737 | 0.178 | 0.699 | 0.496 |
| MSTD | 0.72 | 0.17 | 0.671 | 0.477 |
| TopDom | 0.825 | 0.209 | 0.788 | 0.556 |
| deepTAD | **0.856** | **0.246** | **0.83** | **0.602** |
| CATAD | 0.749 | 0.175 | 0.703 | 0.5 |
| EMTAD | 0.491 | 0.119 | 0.43 | 0.38 |

**Supplementary** **Table S19.** Boundary tagged ratio around the TAD boundaries (25 kb) for five related biological pieces of evidence via KR-normalized Hi-C data on chr1-X of HIC074 at 50kb resolution in the K562 cell line.

|  | CTCF | HK genes | RAD21 | SMC3 | TSS |
| --- | --- | --- | --- | --- | --- |
| deDoc | 0.835 | 0.217 | 0.685 | 0.706 | 0.64 |
| MSTD | 0.832 | 0.195 | 0.635 | 0.666 | 0.617 |
| TopDom | **0.898** | 0.252 | 0.76 | 0.785 | 0.691 |
| deepTAD | 0.897 | **0.264** | **0.775** | **0.799** | **0.702** |
| CATAD | 0.883 | 0.233 | 0.72 | 0.74 | 0.673 |
| EMTAD | 0.719 | 0.171 | 0.442 | 0.497 | 0.564 |

**Supplementary** **Table S20.** Boundary tagged ratio around the TAD boundaries (25 kb) for five related biological pieces of evidence via KR-normalized Hi-C data on chr1-X of HIC074 at 25kb resolution in the K562 cell line.

|  | CTCF | HK genes | RAD21 | SMC3 | TSS |
| --- | --- | --- | --- | --- | --- |
| deDoc | 0.699 | 0.151 | 0.518 | 0.545 | 0.461 |
| MSTD | 0.694 | 0.145 | 0.478 | 0.517 | 0.43 |
| TopDom | 0.733 | 0.179 | 0.548 | 0.591 | 0.477 |
| deepTAD | **0.787** | **0.2** | **0.631** | **0.669** | **0.536** |
| CATAD | 0.734 | 0.158 | 0.533 | 0.568 | 0.481 |
| EMTAD | 0.467 | 0.099 | 0.224 | 0.269 | 0.328 |

**Supplementary Table S21.** The number of nested TAD of deepTAD and deDoc on chromosome 20 in five cell lines.

|  | IMR90 | K562 | NHEK | HMEC | HUVEC |
| --- | --- | --- | --- | --- | --- |
| deDoc | 197/367 | 146/245 | 200/344 | 210/368 | 133/238 |
| deepTAD | 17/194 | 39/229 | 23/205 | 16/163 | 35/220 |

Values in the table are expressed as Number of nested TADs/Number of total TADs.

**Supplementary Table S22.** Effect of different levels of Gaussian noise on model performance.

| noise ratio | precision | recall | f1-score |
| --- | --- | --- | --- |
| 0.003 | 0.905±0.01 | 0.9±0.008 | 0.903±0.001 |
| 0.005 | 0.905±0.008 | 0.898±0.006 | 0.902±0.001 |
| 0.007 | 0.899±0.01 | 0.903±0.009 | 0.901±0.001 |

**Supplementary Table S23.** Evaluation of cosine similarity and Euclidean distance for detecting nested TADs on chromosomes 20-22 of HIC002 at 25kb resolution using TADadjR².

|  | chr20 | chr21 | chr22 |
| --- | --- | --- | --- |
| Euclidean distance | 0.765 | 0.655 | 0.631 |
| Cosine similarity | 0.773 | 0.672 | 0.645 |
